# The Role of Diffusion Tensor Tractography in Assessment of Spondylotic Myelopathy

**DOI:** 10.7759/cureus.25778

**Published:** 2022-06-09

**Authors:** Ramesh Akshiitha J, Ganesan Gopinath, Moorthy Divya, Natarajan Paarthipan

**Affiliations:** 1 Radiology, Velammal Medical College Hospital and Research Institute, Madurai, IND; 2 Radiology, Panimalar Medical College Hospital and Research Institute, Chennai, IND; 3 Radiology, Saveetha Medical College and Hospital, Chennai, IND

**Keywords:** magnetic resonance imaging (mri), diffusion tensor tractography (dtt), diffusion-weighted imaging (dwi), spondylotic myelopathy, cervical spondylosis

## Abstract

Introduction

Cervical spondylotic myelopathy (CSM) describes a neurological deficit related to the spinal cord due to the changes in the facet joints and discs of the cervical spine as a result of degeneration. Diagnosis is mainly dependent on imaging. Diffusion tensor tractography (DTT), being a non-invasive technique, shows better sensitivity when compared to the conventional T2WI sequence in the early detection of cervical spondylotic myelopathy (CSM). The objective was to determine the diagnostic accuracy of the apparent diffusion coefficient (ADC) in predicting high T2 signals in CSM.

Methods

A prospective observational study was done on 47 subjects aged between 25 and 70 years, referred to the department of radiology with clinical and imaging evidence of CSM in a tertiary care institute in Chennai. Nurick classification system was used to assess severity clinically. Diffusion-weighted imaging and DTT were done with 1.5 Tesla MRI. The primary outcome variable was a high T2 signal. Mean fractional anisotropy (FA) at the stenotic level and ADC value at a stenotic level were considered explanatory variables. The sensitivity, specificity, predictive values, and diagnostic accuracy of the screening test with the decided cut-off values along with their 95% CI were presented. P-value <0.05 was considered statistically significant. SPSS version 22 (IBM Inc., Armonk, New York) was used for statistical analysis.

Results

The mean age was 48.26 ± 10.28 years. The majority (72.34%) were males, the majority (42.55%) had a Nurick score of two, and 25.53% had a Nurick score of one. Twenty-six (55.32%) reported a high T2 signal, 36 (76.60%) had elevated ADC, and 11 (23.40 %) had no elevated ADC. There was a statistically significant difference in mean FA and ADC values across groups categorized as non-stenotic level and stenotic level (p-value <0.05). The ADC value had a moderately high sensitivity (76.92%) and low specificity (23.81%) in predicting high T2 signals with a diagnostic accuracy of 53.19%.

Conclusion

DTI parameters at stenotic level (ADC and FA values) in patients with cervical spondylosis help in the early detection of cervical cord compressive myelopathy prior to the appearance of T2 signal changes in conventional MRI, thereby improving clinical outcome and patient management.

## Introduction

Cervical spondylosis is a commonly occurring degenerative condition of the aging spinal cord [1]. It comprises an extensive range of degenerative changes involving the components of a cervical spine like intervertebral discs, joints, and laminae. It is characterized by the formation of bone spurs, protrusion of intervertebral discs, narrowed cervical canals, and desiccation of intervertebral discs [2]. Cervical spondylotic myelopathy (CSM) describes a neurological deficit related to the spinal cord due to the changes in the facet joints and discs of the cervical spine as a result of degeneration [3]. It is usually characterized by neck pain, pain in the arms, numbness, weakness, stiffness, and headache [1, 3, 4].

Globally, neck pain is a common symptom [5]. Among the leading cause of disability-adjusted life years (DALYs), low back pain and neck pain were the fourth leading cause globally in 2015 [6, 7]. The commonly reported types of prevalence for neck pain were one year (39%), point (13%), lifetime (13%), six months (11%), one month (10%), and one week (10%) [8]. A community-based cross-sectional study done in china on 3859 adults showed that the prevalence of cervical spondylosis was 13.76%. It was higher in females (16.51%) compared to males (10.49%). The age group between 45 to 60 years had the highest prevalence [9]. Cervical spondylosis not only affects the life quality but also increases the economic burden since open surgery is a regular treatment method. The basic and initial method for diagnosing compressive myelopathy in a symptomatic patient is conventional magnetic resonance imaging (MRI) of the spine, T2-weighted (T2W) sequence. Although conventional MRI is considered the imaging modality of choice, it does not correlate with clinical findings at times and lags in predicting early spondylotic myelopathy. There is an apparent disconnect between heterogenous clinical presentation in CSM and MRI findings. It is due to the lack of the ability of conventional MRI to highlight microstructural spinal cord changes in CSM. To address this concern, diffusion-weighted imaging (DWI) has been increasingly used for studying cord microstructure in patients with CSM [10]. Its diagnosis is based primarily on clinical manifestations and imaging evidence. On T2-weighted MR image, the compressed part of the spinal cord demonstrates a specific high-intensity signal [11]. But, the sensitivity of T2-weighted imaging alone is low for detecting subtle structural changes of the cord in myelopathy, particularly in subjects with chronic onset of symptoms [12, 13]. Hence, there is difficulty in evaluating the condition of the compressed spinal cord with such imaging modalities. Many patients with severe clinical symptoms showed normal cord signals, which proved the disparity between clinical severity and T2 hyper-intensity. Therefore, there is a need to identify an alternative for early and accurate detection of spinal cord changes. Diffusion tensor imaging (DTI), with its ability to detect microstructural changes in the spinal cord, can become an alternative or adjunct for T2W image (T2WI) [14, 15]. Diffusion tensor tractography (DTT) is an application of MRI diffusion tensor imaging, a non-invasive technique which measures the random motion of water molecules, thereby providing information about cellular integrity. DTT is used to visualize the fiber tract pathways in vivo and can provide a three-dimensional presentation of white matter diffusion in the direction of fibers (axons). Hence the present study was carried out with the primary objective of determining the diagnostic accuracy of the apparent diffusion coefficient (ADC) and fractional anisotropy (FA) values at the stenotic level obtained by DTT in predicting a high T2 signal in CSM. The secondary objective was to correlate the clinical severity graded by the Nurick classification system with DTT and T2 weighted MRI findings.

## Materials and methods

A prospective observational study was done on 47 subjects referred to the department of radiology with complaints of cervical myelopathy in a tertiary care institute in Chennai. After getting clearance from the ethical committee, the data collection was done between November 2017 to January 2018. Informed consent was obtained from all the subjects after explaining in detail the objectives of the study, procedure, risks, and benefits involved. Subjects aged between 25 to 70 years with a clinical diagnosis of spondylotic myelopathy and evidence of spondylosis documented by conventional radiography or CT or MRI were included in the study. Subjects with h/o radiculopathy with known T2 signal changes in conventional MRI, subjects with a history of spine surgery or spinal injury, or any other known neurological disease were excluded from the study. Subjects with contraindication for MRI (including claustrophobia) were also excluded from the study.

Each subject referred to the department of radiology for spondylotic myelopathy had been evaluated and scored by the clinicians using the Nurick classification system for myelopathy. They were assigned a specific severity score ranging from one to five, with five being the highest level of dysfunction.

They were radiologically evaluated using the following MRI protocol: Sagittal T1-weighted sequences, Sagittal T2-weighted sequences, Axial T2-weighted sequences, and Sagittal diffusion tensor imaging.

All examinations were carried out in a 1.5 tesla MRI machine (Multiva; Philips Healthcare, Amsterdam, Netherlands) in patients selected according to the inclusion criteria. The diffusion tensor imaging acquisition was based on single-shot echo-planar imaging (SSEPI) and included diffusion-weighted, FA mapping, ADC mapping, trace weighted, tensor, and mosaic. Abnormal T2W or T1W spinal cord signal presence was analyzed. Using a post-processing 3D software program, diffusion tensor imaging (DTI) was obtained. Artefactual voxel corresponding to noise was determined and removed. The ADC and FA map was then calculated. Then DTI values from the FA and ADC map were acquired using the region of investment (ROI) method by drawing a circle on tensor images. Analysis was done by the radiologist with prior information about the patients' clinical information.

The primary outcome variable was a high T2 signal. Mean FA at the stenotic level and ADC value at a stenotic level were considered explanatory variables. Nurick score was another relevant variable considered for analysis. Descriptive analysis was carried out by mean and standard deviation for quantitative variables and frequency and proportion for categorical variables. Data was also represented using appropriate diagrams like bar diagrams, pie diagrams, and box plots. The association between categorical explanatory variables and the quantitative outcome was assessed by comparing the mean values. The mean differences along with their 95% CI were presented. The correlation between the Nurick score and FA and ADC values at the stenotic level was assessed by plotting the data in a scatter diagram and assessing the Spearman rank-order correlation coefficient (Spearman Rho). A high T2 signal was considered the gold standard. ADC was considered a screening test. The utility of ADC values and FA at a stenotic level in predicting high T2 signal on MRI was assessed by receiver operative curve (ROC) analysis. The area under the ROC curve, along with its 95% CI and p-value, were represented. The sensitivity, specificity, predictive values, and diagnostic accuracy of the screening test with the decided cut-off values along with their 95% CI were presented. The reliability of the screening test was assessed by kappa statistics along with its 95% CI and p-value. A p-value of <0.05 was considered statistically significant. Data was analyzed by using SPSS software version 22 (IBM Inc., Armonk, New York) [16].

## Results

A total of 47 subjects were included in the final analysis. The mean age was 48.26 ± 10.28 years. The majority (72.34%) were males and 27.66% were females. Out of 47 participants, the majority (42.55%, 20 out of 47) had a Nurick score of two, and 25.53% had a Nurick score of one. Out of 47, 26 (55.32%) reported a high T2 signal. 36 (76.60%) had elevated ADC, and 11 (23.40 %) had no elevated ADC (Table 1).

**Table 1:** Summary of baseline parameters ADC - apparent diffusion coefficient, FA - fractional anisotropy

Parameters	Summary
Age	48.26 ± 10 .28 (range 25 to 68)
Gender	
Male	34 (72.34%)
Female	13 (27.66%)
Nurick score	
1	12 (25.53%)
2	20 (42.55%)
3	8 (17.02%)
4	5 (10.64%)
5	2 (4.26%)
High T2 signal	26 (55.32)
Elevated ADC	
Elevated ADC (Stenotic > non-stenotic)	36 (76.60%)
No elevated ADC (Stenotic value ≤ non-stenotic level)	11 (23.40%)
FA value at stenotic level (decreased)	47 (100%)

Sample images of a case

**Figure 1:**
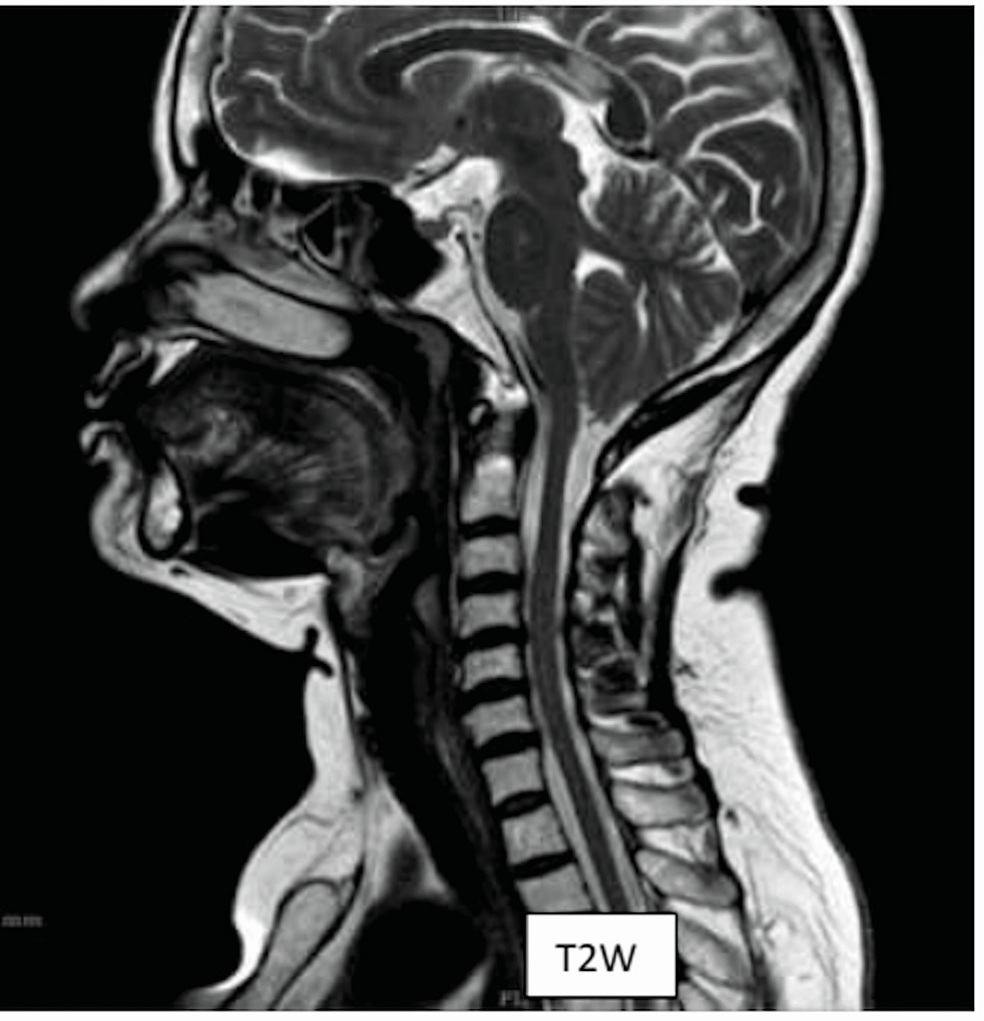
T2 sagittal image showing spondylosis at the C4-5 level with normal T2 cord signals

**Figure 2:**
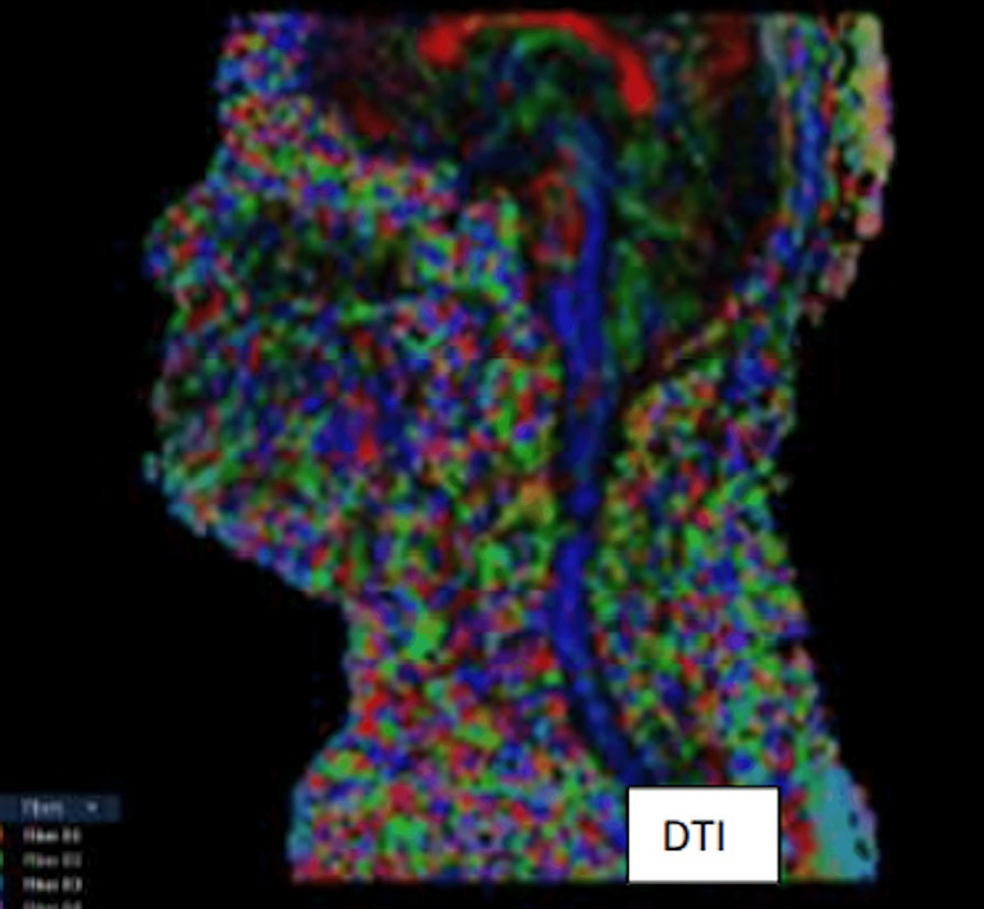
DTI Image showing spinal cord fiber DTI - diffusion tensor imaging

**Figure 3:**
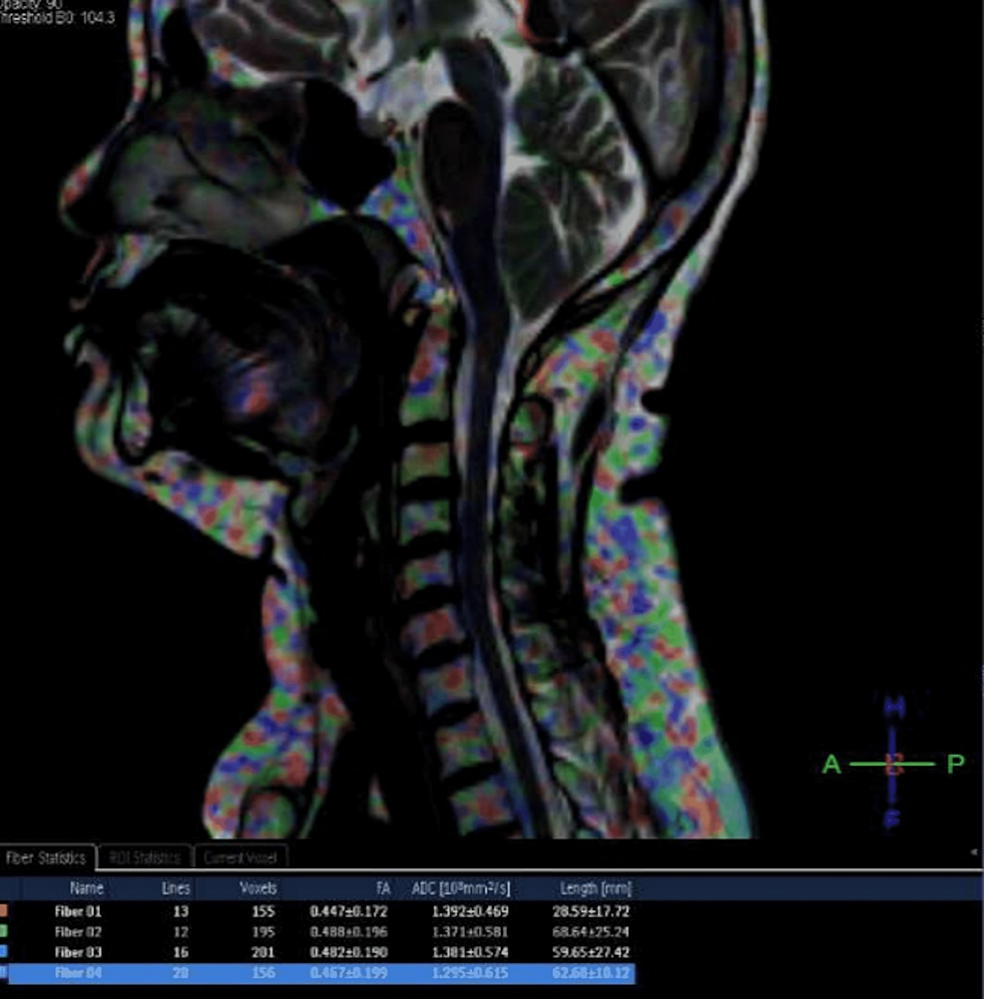
DTI fusion image showing decreased FA and increased ADC values at the C4-5 stenotic level (fiber 1) when compared to other levels DTI - diffusion tensor imaging, FA - fractional anisotropy, ADC - apparent diffusion coefficient

T2 image (Figure 1) showing spondylosis & normal T2 signals of cervical cord at the C4-5 level. DTI images (Figure 2, 3) showing decreased FA and increased ADC values (fiber 1) at the C4-5 level in the cervical cord when compared to other levels.

There was a statistically significant difference between mean FA and ADC values across groups categorized as non-stenotic level and stenotic level (p-value <0.05) (Table 2).

**Table 2:** Comparison of mean of FA and ADC value between non-stenotic level and stenotic level (N=47) FA - fractional anisotropy, ADC - apparent diffusion coefficient

	Non-stenotic level	Stenotic level	Mean difference	p-value
Fractional anisotropy (FA)	0.78 ± 0.05	0.49 ± 0.13	0.29	<0.001
Apparent diffusion coefficient (ADC)	1.03 ± 0.14	1.20 ± 0.16	0.17	<0.001

**Figure 4:**
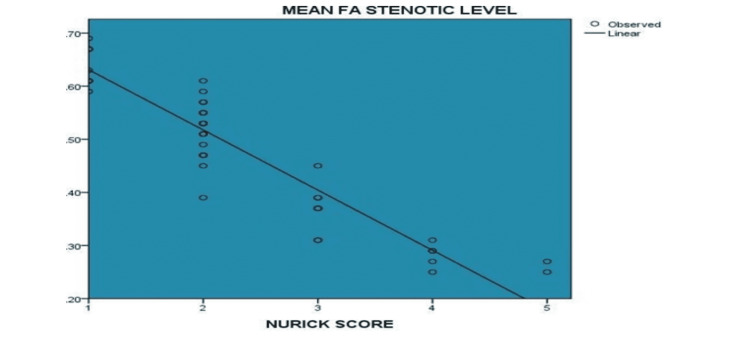
Correlation between the Nurick score and FA at stenotic level (N= 47) FA - fractional anisotropy

There was a strong negative correlation (Spearman’s rho value of -0.938 and a p-value of <0.001) between the fractional anisotropy (FA) value at stenotic level and the Nurick clinical score (Figure 4).

**Figure 5:**
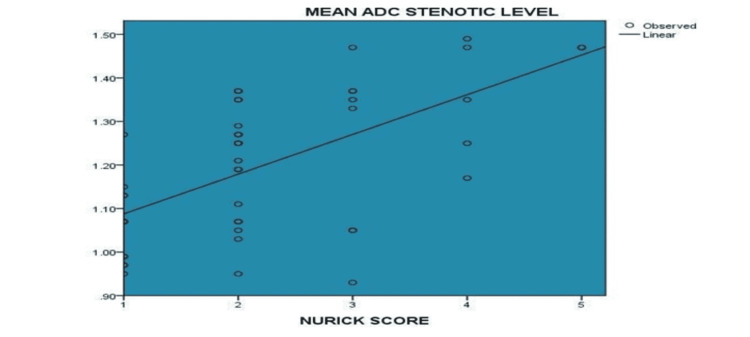
Correlation between Nurick score and ADC at stenotic level (N=47) ADC - apparent diffusion coefficient

There was a moderately positive correlation (Spearman’s rho value of 0.564 and a p-value of <0 .001) between the Nurick score and mean ADC value at the stenotic level (Figure 5).

Increasing Nurick scores like 4 and 5 were reported more commonly in high T2 signal patients. 80% of subjects with a Nurick score of 4 and 100% of subjects with a score of 5 had a high T2 signal. The difference in elevated and non-elevated ADC across the intensity of the T2 signal was statistically insignificant. with a p-value of 0.953 (Table 3).

**Table 3:** Association of a high T2 signal with the Nurick score and elevated ADC of the study population (N=47) *No statistical test was applied due to 0 subjects in the cells, ADC - apparent diffusion coefficient

	High T2 signal		p-value
	Yes	No	
Nurick score			
1	4 (33. 33 %)	8 (66. 67%)	*
2	12 (60 %)	8 (40%)	
3	4 (50 %)	4 (50%)	
4	4 (80 %)	1 (20%)	
5	2 (100 %)	0 (0%)	
	Yes (N= 26)	No (N=21)	
ADC			
Elevated ADC	20 (76 .92%)	16 (76.19%)	0.953
No elevated ADC	6 (23. 08%)	5 (23. 81%)	

The ADC value had a sensitivity of 76.92% in predicting a high T2 signal. Specificity was 23.81%, the false-positive rate was 76.19%, and the false-negative rate was 23.08%. The positive predictive value was 55.56%, negative predictive value was 45.45%, and the total diagnostic accuracy was 53.19% (Table 4).

**Table 4:** Predictive validity of ADC in predicting a high T2 signal (N=47) ADC - apparent diffusion coefficient

Parameter	Value	95% CI	
		Lower	Upper
Sensitivity	76.92%	56.35%	91.03%
Specificity	23.81%	8.22%	47.17%
False-positive rate	76.19%	52.83%	91.78%
False-negative rate	23.08%	8.97%	43.65%
Positive predictive value	55.56%	38.10%	72.06%
Negative predictive value	45.45%	16.75%	76.62%
Diagnostic accuracy	53.19%	38.08%	67.89%

## Discussion

Cervical spondylosis is a commonly occurring degenerative condition of the aging spinal cord [1]. It comprises an extensive range of degenerative changes involving the components of a cervical spine like intervertebral discs, joints, and laminae. As age progresses, degenerative changes like disc desiccation, bulge, and osteophyte formation cause chronic compression of the spinal cord. Cervical spondylosis might become a public health concern. The diagnosis of CSM is constructed upon a combination of conventional MRI findings and clinical findings. Primary evaluation of degenerative changes can be detected using a plain cervical spine X-ray. CT myelography can be done for patients with metallic implants where MRI is contraindicated. Though conventional MRI is the best modality to assess CSM, it is limited by many factors, including acquisition parameters, field strength, the water content of the spinal cord, and others. Thus, the changes in the white matter fiber tracts or the integrity of the spinal cord tracts cannot be assessed. MRI is non-invasive diagnostic imaging that can evaluate the spinal cord in axial, coronal, and sagittal planes and also spinal canal narrowing. It can also detect myelomalacia changes due to cervical spondylotic myelopathy. But CT is considered superior in evaluating spinal canal narrowing, as MRI has limitations in evaluating osteophytes and bony structures.

The mean age in the present study was 48.26 ± 10.28 years. The majority (72.34%) were males. There was a statistically significant difference in mean FA and ADC values across groups categorized as non-stenotic level and stenotic level (p-value <0.05). A study by Banaszek et al. [14] showed that DTI could reveal spinal cord impairment in early-stage cervical spondylosis subjects before the visibility of those changes on conventional MRI scans. It was revealed by their study on 132 symptomatic subjects with varying degrees of cervical spondylosis and 25 controls. They found significant differences in fractional anisotropy values between the control subjects and subjects with cervical spondylosis, including early-stage patients who did not yet show spinal cord compression on plain MRI scans. They concluded that the mean fractional anisotropy values were significantly associated with the anteroposterior diameter of the spinal canal and with space available for the spinal cord index. FA values can help in the early detection of cervical cord compressive myelopathy before the appearance of changes in conventional MRI, thereby improving the clinical outcome and helping in deciding on treatment plans [15]. The manifestation in T2WI is a high signal inside the cord. The T2WI sign appears late in subjects with chronic cervical spondylosis and also has low sensitivity for detecting spinal cord myelopathies [17].

DTI has better sensitivity in comparison with conventional T2WI sequence in the early detection of CSM as it shows abnormalities in the spinal cord before the development of T2 hyperintensity in patients with CSM [18]. Conventional MRI is less sensitive in comparison with DTI in detecting subtle pathological changes of the spinal cord [19]. The FA parameter shows higher sensitivity and specificity among the various DTI parameters for early detection of spinal cord subtle abnormalities in comparison with conventional T2WI [17]. In CSM, the damage caused to the spinal tracts is not of the same degree, and the abnormalities of different DTI parameters might show focal or extensive myelopathic changes [20].

A meta-analysis was done involving fourteen studies, matching a total of 479 CSM subjects with 278 controls. Meta-analysis of the most compressed levels demonstrated that FA was significantly reduced (p<0.001) and ADC was significantly increased (p<0.001), demonstrating a statistically significant reduction of FA and increase of ADC in CSM subjects in comparison with healthy subjects [21].

In our study, increasing Nurick scores like 4 and 5 were reported more commonly in high T2 signal patients. Eighty percent of subjects with a Nurick score of 4 and 100% of subjects with a score of 5 had high T2 signals. The difference in elevated and non-elevated ADC across the intensity of the T2 signal was statistically insignificant, with a p-value of 0.953. In our study, the majority (42.55%) had a Nurkc score of 2. Twenty-six people (55.32%) reported a high T2 signal, 36 people (76.60%) had elevated ADC, and 11 people (23.40 %) had no elevated ADC. In our study, a strong negative correlation was noted between FA value at the stenotic level and Nurick clinical score. This was in concordance with similar studies by Jones et al. and Budzik et al. [22, 23]. In our study, a moderate positive correlation was noted between the Nurick score and the mean ADC value at the stenotic level. This was in concordance with similar studies by Demir et al. [24]. In our study, no significant correlation was noted between the Nurick score and a high T2 signal at the stenotic level. These results were in concordance with similar study by Fernández de et Rota J et al. [25]. Our study is also in accordance with Budzik et al. study, which indicates that FA was more sensitive. In their study, FA was reduced in stenotic levels in 100% of the subjects than conventional T2W imaging in the assessment of the severity of CSM and prognosis because high T2 signal intensity does not correlate with DTI parameters or clinical assessment [23]. ADC value had a moderately high sensitivity (76.92%) and low specificity (23.81%) in predicting high T2 signals with a diagnostic accuracy of 53.19% in the present study.

There is an apparent disconnect between heterogeneous clinical presentation in CSM and conventional MRI findings due to the lack of MRI to highlight microstructural spinal cord changes in CSM. Hence, DWI has been and has become increasingly used for studying the cord microstructure in patients with CSM.

Limitations

The hospital-based recruitment of study subjects is a major limitation of the study. It affects the generalizability of the study results.

## Conclusions

DTI parameters at stenotic level (ADC and FA values) in patients with cervical spondylosis help in the early detection of cervical cord compressive myelopathy prior to the appearance of T2 signal changes in conventional MRI, thereby improving clinical outcome and patient management. DTI parameters at the stenotic level have the potential to be used as a tool of investigation for CSM, which requires further evidence for implementation in day-to-day practice.
